# SOX9 is a critical regulator of TSPAN8-mediated metastasis in pancreatic cancer

**DOI:** 10.1038/s41388-021-01864-9

**Published:** 2021-06-23

**Authors:** Junjian Li, Xiaoliang Chen, Liqun Zhu, Zhenghong Lao, Tianhao Zhou, Lijuan Zang, Weiyu Ge, Mengyi Jiang, Jingxuan Xu, Yuan Cao, Shaoqian Du, Yue Yu, Guangjian Fan, Hongxia Wang

**Affiliations:** 1 Shanghai Key Laboratory of Molecular Imaging, Shanghai University of Medicine and Health Sciences, Shanghai, China; 2 State Key Laboratory of Oncogenes and Related Genes, Department of Oncology, Shanghai General Hospital, Shanghai Jiao Tong University School of Medicine, Shanghai, China; 3 The Center for Chronic Disease Control and Prevention, Shenzhen Guangming District Centers for Disease Control and Prevention, Shenzhen, China; 4 Department of Oncology, Liyang People’s Hospital, Liyang, China; 5 Department of Oncology, Deqing People’s Hospital, Huzhou, China; 6 Pathology Center, Shanghai General Hospital, Shanghai Jiao Tong University School of Medicine, Shanghai, China; 7 Department of Medical Oncology, Shanghai Jiaotong University Affiliated Sixth People’s Hospital East Campus, Shanghai, China; 8 Shanghai Experimental School, Shanghai, China; 9 Translational Medicine Center, Shanghai General Hospital, Shanghai Jiao Tong University School of Medicine, Shanghai, China

**Keywords:** Biomarkers, Prognostic markers

## Abstract

Pancreatic ductal adenocarcinoma (PDAC) is the deadliest cancer mainly owing to its proclivity to early metastasis and the lack of effective targeted therapeutic drugs. Hence, understanding the molecular mechanisms underlying early invasion and metastasis by PDAC is imperative for improving patient outcomes. The present study identified that upregulation of TSPAN8 expression in PDAC facilitates metastasis in vivo and in vitro. We found SOX9 as a key transcriptional regulator of *TSPAN8* expression in response to EGF stimulation. SOX9 modulation was sufficient to positively regulate endogenous expression of TSPAN8, with concomitant in vitro phenotypic changes such as loss of cell–matrix adherence and increased invasion. Moreover, increased SOX9 and TSPAN8 levels were shown to correlate in human pancreatic cancer specimens and downregulated in vitro by EGFR tyrosine kinase inhibitors. High expression of SOX9 and TSPAN8 has been associated with tumor stage, poor prognosis and poor patient survival in PDAC. In conclusion, this study highlights the importance of the EGF-SOX9-TSPAN8 signaling cascade in the control of PDAC invasion and implies that TSPAN8 may be a promising novel therapeutic target for the treatment of PDAC.

## Introduction

Pancreatic ductal adenocarcinoma (PDAC) is an extremely lethal cancer worldwide with limited therapeutic options and a dismal prognosis. By 2030, PDAC is poised to become the second primary cause of cancer-related death [1, 2]. Metastasis is the leading cause of mortality and a major driver behind the lethal nature of PDAC [3]. Clinically, most patients are diagnosed at a very late stage with metastatic dissemination, at which point the 5-year survival rate is only 3% [4]. Even in those patients who have had curative surgical resection with clear tumor margins (R0), 75% die of recurrence and metastasis within 5 years after operation [5]. The molecular mechanisms involved in the metastatic cascade remain incompletely understood owing to the complexity of the process. Deep molecular insights into the metastatic process of PDAC are imperative for the development of effective targeted treatment strategies.

Tetraspanins are a superfamily of transmembrane proteins containing four highly hydrophobic transmembrane domains (TMs) and N-terminal and C-terminal cytoplasmic tails [6]. In mammals, the tetraspanin family consists of 33 members, including clusters of differentiation-related protein 9 (CD9), CD37, CD63, CD81 and tetraspanin-8 (TSPAN8; encoded by the gene *TSPAN8*). TSPAN8 has been implicated in many cellular functions because it forms tetraspanin-enriched microdomains (TEMs) with different molecular chaperonins, such as cluster of differentiation (CD) proteins, including CD9, CD37, CD53, CD63 and CD81 [7], integrins, MHC class II antigens and T-cell receptors [8, 9]. Recent evidence suggests that TSPAN8 has an important role in tumor invasion and metastasis in multiple types of tumors, including PDAC [10], ovarian carcinoma [11], gastric adenocarcinoma [12], colon adenocarcinoma [13], liver hepatocellular carcinoma [14], esophageal carcinoma [15], melanoma and glioma [16]. Margot Zöller et al. indicated that TSPAN8 can form a complex with CD49c, CD9 and CD151 to promote cell migration by internalization [17] or can interact with integrin α6β4 [18]. TSPAN8 is a component of exosomes and increases sensitivity and specificity by mediating the reprogramming of target cells [19–21]. TSPAN8 can mediate mesenchymal–epithelial transition by upregulating E-cadherin and downregulating Twist, P120-catenin and β-catenin [22]. Our previous research revealed that the expression level of TSPAN8 is upregulated in breast cancer stem cells and correlates with chemotherapeutic resistance and poor prognosis [8]. Despite its well-established importance in tumor progression, metastasis and drug resistance, most previous studies have focused on how TSPAN8 interacts with other molecules and organizes membrane networks as a ‘molecular facilitator' to achieve its biological functions [23]. However, why and how TSPAN8 expression is switched on during tumor progression are ill-defined. Identifying factors influencing TSPAN8 expression, which enables acquisition of an invasive phenotype, is crucial to improving patient treatment and outcome.

Sex-determining region Y-related high-mobility group (HMG)-box (SOX) proteins are a family of transcription factors (TFs) highly expressed in multiple aggressive tumors [24]. Of the SOX family members, SOX9 is a vital TF characterized by the existence of a SRY box, a 79-amino-acid motif that encodes a conserved HMG DNA-binding domain [25]. Initial studies show that SOX9 participates in the maintenance of stemness in stem/progenitor cells and participates in relevant roles in organogenesis, such as progenitor differentiation, sex determination, oligodendrocyte development and neural crest cell development [26–29]. Recent evidence indicates that SOX9 participates in cancer initiation and tumourigenicity via its regulation of initiating cells, which are functionally linked with TGFβ/Smad, Notch and Wnt/β-catenin signaling activation [30–32]. Clinically, high SOX9 expression is correlated with metastasis, chemoresistance and poor prognosis in multiple tumors [33–39]. In the pancreas, SOX9 is a main regulator of pancreatic progenitor cells and has a vital role in pancreatic endocrine and ductal cell differentiation during pancreas development [39, 40]. Under chronic inflammation, the overexpression of SOX9 induced by epidermal growth factor (EGFR) signaling is indispensable for acinar to ductal metaplasia (ADM) transdifferentiation [41] and for the KRAS-induced initiation of intraepithelial neoplasias [42, 43].

In this study, we demonstrated that upregulation of TSPAN8 expression in PDAC promotes metastasis in vivo and in vitro. We identified SOX9 as a key transcriptional regulator of *TSPAN8* overexpression in response to EGF stimulation. SOX9 depletion by short hairpin RNA (shRNA) expression completely abrogated the enhanced expression of TSPAN8 at the protein and mRNA levels upon EGF treatment. Moreover, immunohistochemistry (IHC) staining analyses of human PDAC specimens determined the correlation between EGFR, SOX9 and TSPAN8 expression. High-expression levels of SOX9 and TSPAN8 were associated with tumor stage, dismal prognosis and poor survival in PDAC. These findings demonstrate the role of the EGF-SOX9-TSPAN8 signaling cascade during tumor metastasis and highlight the importance of TSPAN8 as a valuable therapeutic target for PDAC treatment.

## Results

### TSPAN8 is highly expressed in PDAC and is associated with progression and poor prognosis

**Fig. 1:**
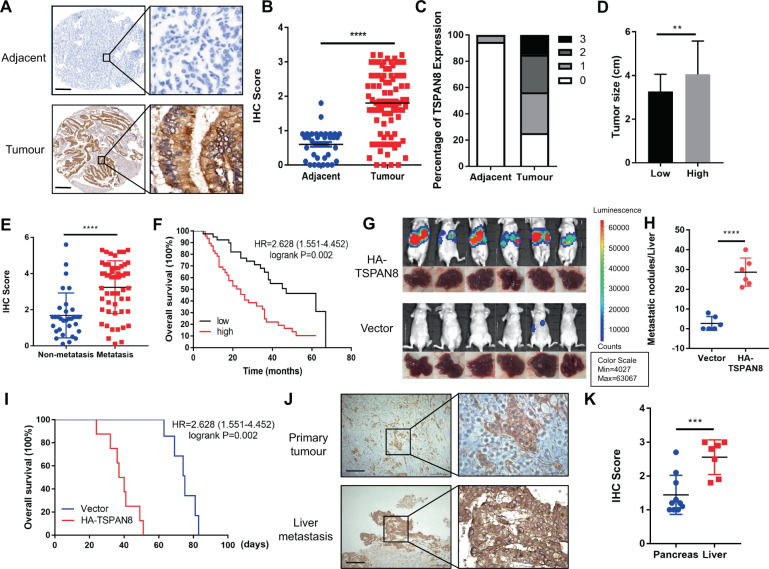
TSPAN8 is highly expressed in PDAC and is associated with progression and poor prognosis. **A**–**C** Immunohistochemical staining (**A**), TSPAN8 expression (**B**–**C**) and tumor size (**D**) data for 87 human pancreatic cancer specimens were analyzed. Representative images of normal adjacent tissues (NATs) and tumor tissues (TTs) are shown. Scale bars: 200 μm. **E** TSPAN8 expression in tumor tissues from patients with distant metastasis and without distant metastasis was analyzed. **F** The survival times of 87 PDAC patients with low (black curve) and high (red curve) TSPAN8 protein levels (low, 47 patients; high, 40 patients) indicated a significant association of the TSPAN8 level with patient survival, as determined by a log-rank test. **G**–**H** A total of 10^6^ SW1990 cells with or without expression of HA-TSPAN8 were injected into athymic nude mice. Representative tumor xenografts are shown (**G**). The number of visible metastatic lesions in the liver was analyzed by a *t* test (**H**). **I** Kaplan–Meier survival analysis was performed. *P* values were calculated by a log-rank test (*N* = 6 mice). **J**–**K** Immunohistochemical staining for TSPAN8 was performed on 17 PDAC patient specimens of primary tumor tissues and liver metastases (**J**). Scale bars: 200 μm. TSPAN8 expression in primary tumor tissues and liver metastasis tissues was analyzed by a *t* test (**K**). **B**, **D**, **E**, **H**, **K** the values are presented as the means ± SDs. *P < 0.05, **P < 0.01, ***P < 0.001 and ****P < 0.0001.

The expression level of TSPAN8 in tumor tissues (TTs) and normal adjacent tissues (NATs) was determined by tissue microarray analysis, including 87 PDAC patients. IHC staining analysis showed that TSPAN8 was significantly highly expressed in TTs compared with NATs (Fig. 1A–C, Supplementary Table 1). TSPAN8 expression levels were positively associated with tumor size (Fig. 1D). To further research the clinical significance of TSPAN8, TSPAN8 expression in PDAC patients with distant metastasis was compared with that in patients without distant metastasis. The results showed that TSPAN8 expression was significantly higher in PDAC patients with metastasis (Fig. 1E). Furthermore, the expression levels of TSPAN8 were negatively correlated with the overall survival time of PDAC patients (*P* = 0.002, Fig. 1F). In breast cancer and liver hepatocellular carcinoma, high expressions of TSPAN8 were observed (Fig S1A, B). In addition, *TSPAN8* mRNA data from the TCGA and GTEx databases were analyzed via the GEPIA online tool (http://gepia.cancer-pku.cn). Consistently, we observed that the level of *TSPAN8* mRNA was significantly enhanced in TTs compared with NATs. Similar phenomena were also observed in colon adenocarcinoma, liver hepatocellular carcinoma, prostate carcinoma, rectal adenocarcinoma, stomach adenocarcinoma and esophageal carcinoma (Fig S1C).

To further assess the impact of TSPAN8 on PDAC metastasis, 10^6^ SW1990 cells with or without HA-TSPAN8 expression were injected into athymic nude mice by intrasplenic injection to construct a liver metastasis mouse model. SW1990 cells expressing HA-TSPAN8 showed a strong metastasis ability (Fig. 1G, H, Fig S1D, E) and shortened survival times (Fig. 1I). In contrast, TSPAN8 depletion reduced liver metastasis and prolonged mouse survival time (data not shown). To determine the clinical relevance of TSPAN8 in PDAC metastasis, we performed IHC staining of primary tumor and liver metastasis tissues from 17 PDAC patients. A significant increase in TSPAN8 expression in liver metastasis tissues compared with pancreatic primary TTs was observed (Fig. 1J, K). These results suggest that TSPAN8 expression is positively correlated with tumor metastasis and poor prognosis in PDAC patients.

### TSPAN8 has a key role in pancreatic tumor cell invasion and migration

**Fig. 2:**
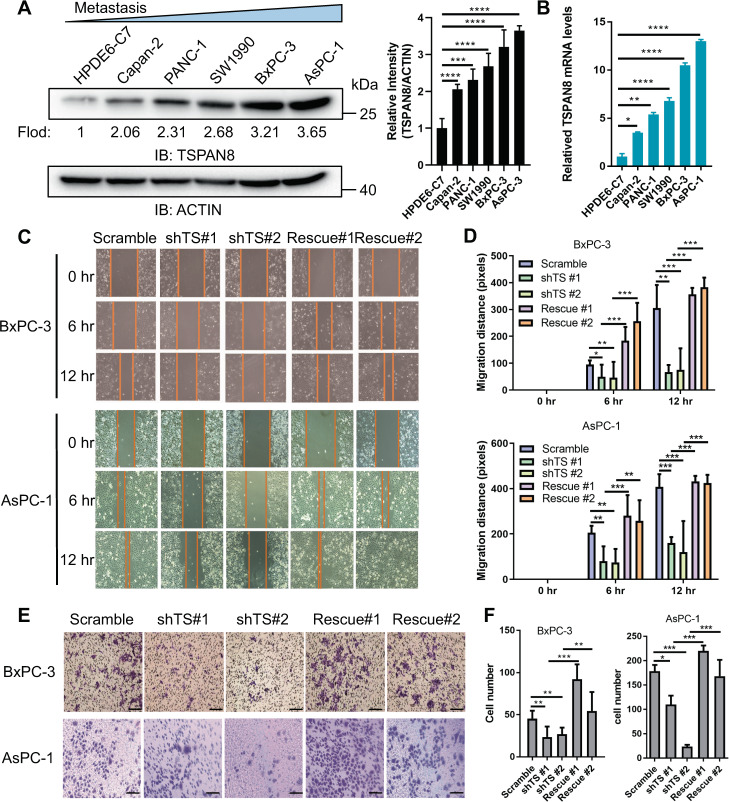
TSPAN8 plays a key role in pancreatic tumor cell invasion and migration. **A**, **B** TSPAN8 expression was examined in the indicated cell lines by immunoblot analysis (**A**) or qPCR (**B**). **C**–**D** A wound-healing assay was performed on BxPC-3 and AsPC-1 cells expressing the control shRNA, *TSPAN8* shRNA vector or reconstituted with shRNA-resistant TSPAN8. **E**–**F** Boyden chamber Matrigel invasion assays were performed on BxPC-3 and AsPC-1 cells expressing the control shRNA, TSPAN8 shRNA vector or reconstituted with shRNA-resistant TSPAN8. Scale bars: 100 μm. **A**, **B**, **D** and **F** the experiments were performed in triplicate, and the *t* test was performed. The values are presented as the means ± SDs. *P < 0.05, **P < 0.01, ***P < 0.001 and ****P < 0.0001.

To determine the biological roles of TSPAN8 in PDAC metastasis, we carried out an immunoblotting analysis of TSPAN8 expression in a panel of pancreatic cancer cell lines with different metastatic potential (Fig. S2A). The results suggested that the expression level of TSPAN8 was significantly elevated in the malignant pancreatic cancer cell lines BxPC-3, AsPC-1 and SW1990 compared with the normal cell line HPDE6-C7 (Fig. 2A). In addition, the expression of TSPAN8 showed a positive relationship with the increasing metastatic ability of the cancer cells. Consistent with the results of the protein expression levels, quantitative PCR analysis showed that *TSPAN8* mRNA levels were significantly upregulated in PDAC cancer cells compared with normal cells (Fig. 2B). Next, functional analysis was performed by cell migration and invasion assays. TSPAN8 depletion by *TSPAN8*-specific shRNA significantly reduced the invasion ability of BxPC-3 and AsPC-1 cells (Fig. 2C–F). To further determine whether this effect resulted from TSPAN8 depletion specifically, BxPC-3 and AsPC-1 cells with TSPAN8 depletion were administered shRNA-resistant TSPAN8 (rescue TSPAN8) (Fig S2B, C). As a result, the expression of rescue TSPAN8 significantly reversed the inhibitory effect of TSPAN8 depletion on cellular invasion (Fig. 2C–F), suggesting that TSPAN8 has an important role in enhancing pancreatic cancer cell invasion. To further evaluate the metastatic functions of TSPAN8, TSPAN8 was transiently overexpressed in HPDE6-C7 cells (Fig S2D). As shown in Fig S2E, F, the cell invasion ability was significantly enhanced by TSPAN8 overexpression. These results demonstrate that TSPAN8 is important for the regulation of cellular invasion and tumor metastasis.

### SOX9 is required for EGF-induced TSPAN8 upregulation

**Fig. 3:**
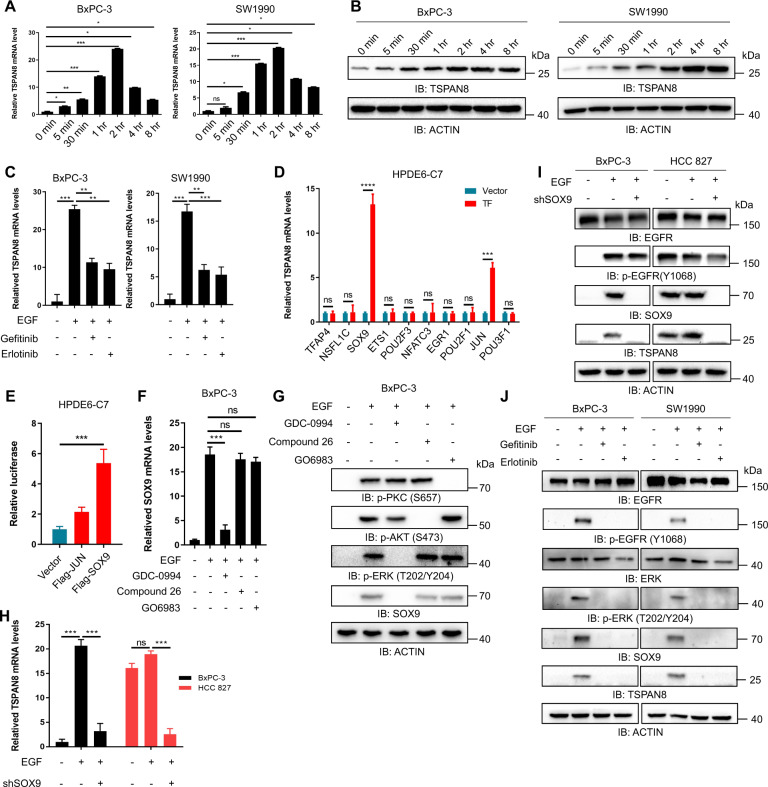
SOX9 is required for EGF-induced TSPAN8 upregulation. **A**, **B** BXPC-3 and SW1990 cells were treated with or without EGF (100 ng/ml) for different times. *TSPAN8* mRNA levels were analyzed by qPCR (**A**), and immunoblot analysis was performed with the indicated antibodies (**B**). **C** BxPC-3 and SW1990 cells were treated with or without gefitinib (10 μM) or erlotinib (10 μM) for 1 hr prior to EGF treatment (100 ng/ml) for 2 hr. Relative mRNA levels were analyzed by qPCR. **D** HPDE6-C7 cells with stable overexpression of different TFs were generated. *TSPAN8* mRNA levels were analyzed by qPCR. **E** HPDE6-C7 cells with stable overexpression of the *TSPAN8* promoter and Flag-SOX9 or Flag-JUN were generated. A luciferase activity assay was performed. **F**, **G** BxPC-3 and SW1990 cells were treated with GDC-0994 (10 μM), compound 26 (10 μM) and GO6983 (10 μM) for 1 hr prior to EGF treatment (100 ng/ml) for 2 hr. The *SOX9* mRNA and protein levels were analyzed by qPCR and immunoblotting with the indicated antibodies, respectively. **H**, **I** BxPC-3 and HCC827 cells with or without stable expression of shSOX9 were treated with EGF (100 ng/ml) for 2 hr. The *TSPAN8* mRNA and protein levels were analyzed by qPCR (**H**) and immunoblotting with the indicated antibodies (**I**). **J** BxPC-3 and SW1990 cells were treated with or without gefitinib (10 μM) or erlotinib (10 μM) for 1 hr prior to EGF treatment (100 ng/ml) for 2 hr. Protein expression was analyzed by immunoblotting with the indicated antibodies. In **A**, **C**–**F**, the experiments were performed in triplicate, and the *t* test was performed. The values are presented as the means ± SDs. *P < 0.05, **P < 0.01, ***P < 0.001 and ****P < 0.0001.

Epidermal growth factor (EGF)/EGFR signaling has been implicated in many steps in the processes of tumor invasion and metastasis. In PDAC, EGFR is overexpressed in more than half of cases [44, 45]. To determine whether upregulation of TSPAN8 expression is a response to EGF, we evaluated the changes in TSPAN8 expression in BxPC-3 and SW1990 cells with or without EGF stimulation. QPCR analysis indicated that EGF stimulation resulted in a dramatic increase in *TSPAN8* mRNA in a time-dependent manner (Fig. 3A). In line with this, the protein expression levels of TSPAN8 were also upregulated upon EGF treatment (Fig. 3B, Fig S3A, B). However, EGF-induced upregulation of *TSPAN8* mRNA expression was suppressed by the EGFR tyrosine kinase inhibitors gefitinib (10 μM), erlotinib (10 μM) and AG1478 (10 μM) in BxPC-3 and SW1990 cells (Fig. 3C, Fig S3C).

*TSPAN8* mRNA expression is regulated by upstream promoters and TFs during tumor progression. To investigate the mechanism underlying *TSPAN8* upregulation, potential TFs of *TSPAN8* were predicted via the JASPAR (http://jaspar.genereg.net) and AliBaba 2.1 (http://gene-regulation.com/pub/programs/alibaba2) online tools. After combined analysis via JASPAR and AliBaba 2.1, 10 TFs were obtained. To assess the potential influence of predicted TFs on *TSPAN8* mRNA expression, HPDE6-C7 cells with overexpression of 10 TFs were generated. Overexpression of SOX9 and JUN significantly enhanced *TSPAN8* mRNA expression, and SOX9 had the greatest influence (Fig. 3D). To further study the underlying relationships, luciferase reporter assays were performed with Flag-SOX9 and Flag-JUN plasmids.

As shown in Fig. 3E, SOX9, but not JUN, caused a significant change in the luciferase activity of the *TSPAN8* promoter-reporter construct, indicating that SOX9 might regulate *TSPAN8* transcription. The mRNA and protein expression levels of SOX9 were further found to increase upon EGF treatment but were reduced by gefitinib and erlotinib treatment in BxPC-3 cells (Fig. 3F, G), suggesting an EGF-dependent regulatory mechanism. To determine the key signaling molecule that mediates SOX9 upregulation, a panel of inhibitors linked to EGFR signaling pathway kinases was used, and we found that SOX9 expression was significantly decreased by treatment with the ERK inhibitor GDC-0994 but not the AKT inhibitor compound 26 or the PKCα inhibitor GO6983 in BxPC-3 cells (Fig. 3F, G), consistent with a previous report [46, 47].

To study the transcriptional regulation of SOX9 to *TSPAN8*, SOX9 depletion by *SOX9*-specific shRNA was performed in BxPC-3 cells (Fig S3D, E). As expected, the results of qPCR and immunoblot analyses showed that the enhanced expression of TSPAN8 at the mRNA and protein levels upon EGF treatment was inhibited by *SOX9*-specific shRNA in BxPC-3 cells (Fig. 3H, I). In addition, HCC827 cells, which have constitutively active EGFR, were treated with or without shSOX9, and the results showed that TSPAN8 was high even without EGF stimulation, but shSOX9 reversed this increase (Fig. 3H, I). To further confirm the EGF-induced upregulation of TSPAN8 expression, BxPC-3 and SW1990 cells were treated with gefitinib, erlotinib or AG1478 under EGF stimulation, and immunoblotting was performed. The results showed that gefitinib, erlotinib and AG1478 inhibited the phosphorylation of EGFR and ERK, which reduced SOX9 expression and reversed the increase in TSPAN8 expression caused by EGF stimulation (Fig. 3J, Fig S3F). These data indicate that SOX9 is required for the upregulation of *TSPAN8* expression mediated by EGF.

### SOX9 regulates *TSPAN8* mRNA expression by directly binding to its promoter

**Fig. 4:**
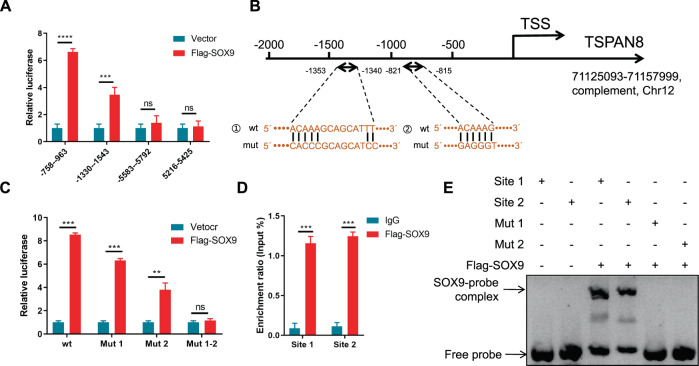
SOX9 regulates *TSPAN8* mRNA expression by directly binding to its promoter. **A** 293 T cells with transient expression of Flag-SOX9 and sites in the *TSPAN8* promoter were cultured for 48 h for a luciferase activity assay. **B** Wild-type (wt) and mutant (mut) binding sites for SOX9 in the *TSPAN8* promoter region. **C** A luciferase activity assay was performed in 293 T cells with transient expression of the wt or mut *TSPAN8* gene promoter and Flag-SOX9. **D** ChIP-qPCR analysis was performed. The *Y* axis shows the value normalized to the input. **E** EMSA was performed with Flag-SOX9 and probes for the site 1, site 2, mut 1 or mut 2 region of the *TSPAN8* promoter. **A**, **C** and **D**, the experiments were performed in triplicate, and the *t* test was performed. The values are presented as the means ± SDs. **P < 0.01, ***P < 0.001 and ****P < 0.0001.

Since SOX9 is known as a TF [48] and our results determined that SOX9 is responsible for the upregulation of *TSPAN8* mRNA expression upon EGF/EGFR activation, we wondered whether SOX9 could transcriptionally regulate *TSPAN8* expression in PDAC. To further investigate this result, chromatin IP sequencing was performed with an anti-Flag antibody in HDPE6-C7 cells stably overexpressing Flag-SOX9, and 10625 different peaks mapping to 1077 genes were obtained (Fig S4A). To examine the functions of these genes, we performed GO term enrichment analysis. The GO terms response to stimulus, cell migration, motility and growth were enriched with these genes, which include EGFR signaling pathway-related genes (Fig S4B, Supplementary Table 2). All enriched GO terms with *P* < 0.05 are summarized in Supplementary Table 3. KEGG pathway enrichment analysis showed that the regulation of the pathways of the actin cytoskeleton and PI3K-AKT signaling were enriched (Fig S4C). Meanwhile, four promoter fragments of *TSPAN8* were identified. We discovered that Flag-SOX9 dramatically enhanced luciferase intensity at residues 758–963 and 1330–1543 upstream of the transcriptional start site (TSS) in the promoter region of *TSPAN8* (Fig. 4A). By comparing the binding motifs of SOX9 and the promoter sequence of *TSPAN8*, we identified two binding sites, namely, 1340–1353 and 815–821, upstream of the TSS of *TSPAN8*, which is consistent with previous results [25] (Fig. 4B, Fig S4D). Thereafter, we found that a SOX9 construct with one of the binding sites mutated had a less powerful effect on increasing the expression of *TSPAN8* than the wild-type SOX9 construct. When the two binding sites were mutated simultaneously, SOX9 was not able to induce any activity of the *TSPAN8* promoter (Fig. 4C). ChIP-qPCR analysis confirmed the binding of SOX9 to *TSPAN8* at specific sites (Fig. 4D). Consistent with this finding, electromobility shift assays (EMSAs) showed that SOX9 binds two sites in the promoter of *TSPAN8* (Fig. 4E). Collectively, these data suggest that SOX9 promotes the transcription of *TSPAN8* by binding to two sites in the promoter of *TSPAN8*.

### TSPAN8 and SOX9 expression levels are correlated, and high-expression levels of both are related to poor prognosis

**Fig. 5:**
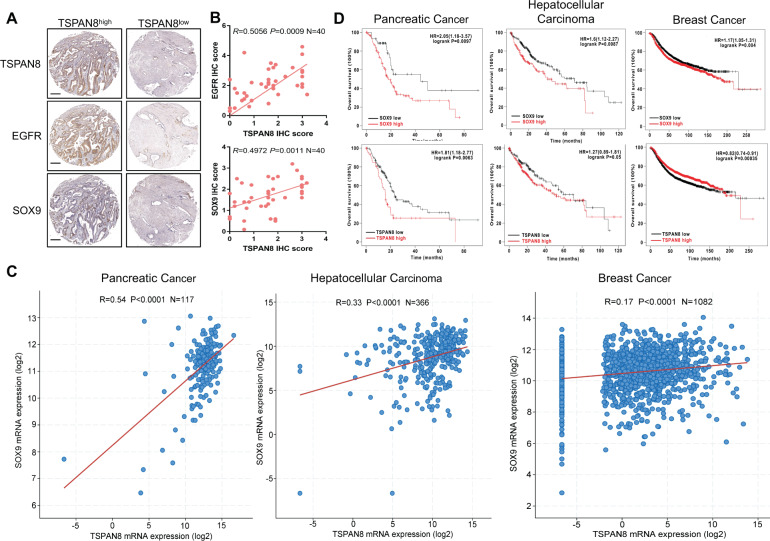
TSPAN8 and SOX9 expression levels are correlated, and high-expression levels of both are related to poor prognosis. **A**, **B** Immunohistochemical staining for TSPAN8, EGFR and SOX9 was performed on 40 human pancreatic cancer specimens. Representative images of TSPAN8 high-expression and TSPAN8 low-expression tumor tissues are shown (**A**). Scale bars: 200 μm. Pearson correlation analysis was performed to evaluate associations between the expression levels of TSPAN8 and SOX9 or TSPAN8 and EGFR (**B**). **C** Correlation analysis of *SOX9* and *TSPAN8* mRNA expression in pancreatic cancer, hepatocellular carcinoma and breast cancer was performed with data from the TCGA data set. **D** Kaplan–Meier survival analysis of patients with pancreatic cancer, hepatocellular carcinoma or breast cancer included in the TCGA data set (the groups were stratified by the SOX9 or TSPAN8 expression level) was performed.

To identify the correlation between the expression levels of TSPAN8, SOX9 and EGFR in PDAC, we performed IHC analysis of an additional cohort of 40 human pancreatic cancer specimens. Pearson correlation analysis depicted a positive association between the expression levels of TSPAN8 and SOX9, as well as between TSPAN8 and EGFR (Fig. 5A, B). Finally, we analyzed the correlation of *SOX9* and *TSPAN8* in the TCGA data set using the cBioPortal online tool (http://www.cbioportal.org). The results showed that the mRNA expression of *TSPAN8* was significantly correlated with *SOX9* in multiple cancers (Fig. 5C, Fig S5A–C). Furthermore, through Kaplan–Meier Plotter (https://kmplot.com/analysis), we found that high-expression levels of SOX9 or TSPAN8 were correlated with poor prognosis in PDAC patients (Fig. 5D). Similar results have also been observed in other cancers, such as liver hepatocellular carcinoma and breast cancer. Collectively, these results strongly suggest that EGF-SOX9 signaling promotes PDAC progression by enhancing *TSPAN8* transcription.

## Discussion

Emerging evidence supports that TSPAN8 has an important role in tumor progression and metastasis [11, 49]. Our previous study demonstrated that TSPAN8 plays a key role in the regulation of breast cancer cell stemness via activation of sonic hedgehog signaling [8]. Here, we demonstrated that TSPAN8 overexpression promoted PDAC cells invasion and migration. Clinically, TSPAN8 expression levels were positively linked with tumor stage, size and axillary node metastasis and inversely correlated with the overall survival time in PDAC patients. Moreover, TSPAN8 overexpression markedly enhanced liver metastasis in vivo and shortened mouse survival time.

**Fig. 6:**
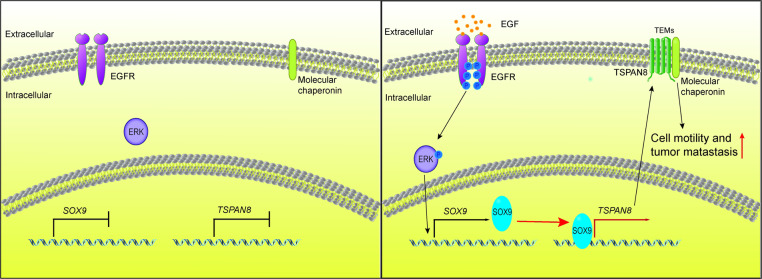
A schematic diagram of the mechanism by which EGF-ERK-SOX9 regulates TSPAN8 to promote tumor metastasis. SOX9 is upregulated upon EGF stimulation and binds two regions in the promoter of the *TSPAN8* gene to upregulate TSPAN8 expression. High TSPAN8 expression leads to enhanced cell invasion. Therefore, in cancer cells, EGF-ERK-SOX9-TSPAN8 signaling enhances tumor metastasis.

Despite the central role of TSPAN8 in critical processes for tumor progression, the underlying molecular mechanisms of TSPAN8 expression upregulation remain poorly understood [50]. In our attempts to investigate the mechanism underlying TSPAN8 overexpression in PDAC cells, we found that SOX9 is a critical regulator of *TSPAN8* transcription. The developmental regulator SOX9 is widely linked to cancer cell proliferation, progression, survival and evasion of senescence in different cancers [39, 51, 52]. In PDAC, SOX9 has oncogenic roles to promote progression and metastasis [41, 53]. In addition, the expression of SOX9 is induced by EGF/EGFR signaling activation [53]. Our results showed that SOX9 expression was upregulated through the EGFR-ERK signaling axis. Given that SOX9 was identified as a potential TF of *TSPAN8* by sequence analysis, we speculated that TSPAN8 overexpression might be caused by SOX9 transcriptional regulation. Immunoblotting and qPCR analyses showed that SOX9 depletion by shRNA completely abrogated the enhanced expression of TSPAN8 induced upon EGF treatment at the protein and mRNA levels. A further mechanistic study indicated that SOX9 bound to two sites of the *TSPAN8* promoter to promote its transcription. Consequently, TSPAN8 expression promoted PDAC invasion and metastasis (Fig. 6). Our analyses of human PDAC specimens depicted a positive correlation among the expression levels of TSPAN8, EGFR and SOX9. Of note, TSPAN8 and SOX9 expression levels were related to poor prognosis in patients with PDAC and other tumors. Taken together, our results illustrate a novel regulatory role of EGF-ERK-SOX9-TSPAN8 signaling in cancer cell migration and invasion.

Our determination of the mechanism by which SOX9 is upregulated under EGF activation and promotes TSPAN8 transcription provides insight for determining how cell motility is regulated in response to environmental stimuli. The EGF/EGFR signaling pathway is frequently deregulated in PDAC. To date, only one targeted drug, erlotinib, which is an orally administered EGFR tyrosine kinase inhibitor, has been approved for the treatment of PDAC. However, its effectiveness has long been questioned due to its low response rate and overall survival rate [54]. This maybe due to the enrichment of TSPAN8 in exosomes. The function of exosomes in tumor metastasis has been widely reported [55]. TSPAN8, as a tetraspanin, form the complexes with other tetraspanin proteins as constitutive components of exosomes to active the signaling cascades in target cells [17, 19–21, 56]. This counteracts the positive response of erlotinib, decreases the overall survival rate and promotes tumor metastasis in PDAC finally. The establishment of the critical role of the EGF-ERK-SOX9-TSPAN8 signaling cascade in promoting tumor metastasis makes TSPAN8 a novel therapeutic target for the treatment of PDAC.

## Materials and methods

### Cell culture

Capan-2, PANC-1, HEK293T and HCC827 cells were cultured in high-glucose Dulbecco’s modified Eagle’s medium (DMEM, HyClone, Illinois, USA) supplemented with 10% fetal bovine serum (FBS, Gibco, New York, USA), 100 U/ml penicillin and 100 mg/ml streptomycin (HyClone, Illinois, USA) at 37 °C with 5% CO_2_. HPDE6-C7, SW1990, AsPC-1 and BxPC-3 cells were cultured in Roswell Park Memorial Institute-1640 medium (HyClone, Illinois, USA) supplemented with 10% FBS, 100 U/ml penicillin and 100 mg/ml streptomycin at 37°C with 5% CO_2_. EGF treatment was administered at a final concentration of 100 ng/ml. All of the cell lines used in this study were purchased from ATCC and routinely tested for mycoplasma contamination every 2 weeks.

### DNA constructs and mutagenesis

For the generation of cell lines with gene depletion or overexpression, the viral skeleton plasmid pLKO.1-Hygro and the EGFP-tagged HA/3xFlag-PGK-Puro vector were transfected into the indicated cell lines to silence and overexpress TSPAN8, respectively. After antibiotic selection, the knockdown efficiency and overexpression efficiency were assessed by immunoblotting. Cells with depleted endogenous TSPAN8 and reconstituted cells with stable expression of shRNA-resistant TSPAN8 (rescue TSPAN8) were utilized for immunoblotting analysis, qPCR assays, cell invasion analysis and tumor xenograft experiments as indicated in our study. pGL3 vectors containing *TSPAN8* WT and mutant promoters were constructed for the luciferase reporter gene assay. The shRNA and PCR primer oligonucleotide sequences used in our research are shown in Supplementary Table 4.

### Materials

Antibodies that recognize TSPAN8 (ab70007, 1:1000), HA (#3724, 1:5000), EGFR (ab52894, 1:8000) and phospho-EFGR Y1068 (ab32430, 1:8000) were purchased from Abcam (Cambridge, UK). Anti-SOX9 (#82630, 1:1000), β-actin (#4970, 1:5000), phospho-p44/42 MAPK (Erk 1/2) T202/Y204, rabbit horseradish peroxidase (HRP)-linked (#7074, 1:5000) and mouse HRP-linked (#7076, 1:5000) antibodies were purchased from Cell Signaling Technology (Massachusetts, USA). Anti-ERK1/2 (16443–1-AP, 1:1000) and phospho-AKT S473 (66444–1-lg, 1:1000) were purchased from Proteintech Group, Inc (Chicago, USA). Anti-phospho-PKCα S657 (sc-377565, 1:100) was purchased from Santa Cruz Biotechnology (Texas, USA).

Recombinant human EGF (rhEGF, PHG0311L) was purchased from Gibco (New York, USA). D-luciferin sodium salt (P1043) was purchased from Promega (Wisconsin, USA).

Gefitinib (HY-50895), Erlotinib (HY-50896), AG1478 (HY-13524), Compound 26 (HY-18296), GDC-0994 (HY-15947) and GO6983 (HY-13689) were purchased from MedChemExpress (MCE) Company (Shanghai, China).

### Transfection

Cells were plated at a density of 1 × 10^6^ per 10 cm dish 24 hours before transfection. Transfection was performed as previously described [8].

### Immunoblotting analysis

Proteins were extracted from cultured cells using RIPA buffer (Beyotime, Shanghai, China) at 4°C followed by immunoblotting with the corresponding antibodies in the presence of protease inhibitor cocktail and phosphatase inhibitor cocktail. The protein concentration was determined using a BCA Protein Assay Kit (Beyotime, Shanghai, China). Proteins from cell lysates were separated by SDS-PAGE, transferred onto polyvinylidene difluoride membranes (Millipore Corporation, Massachusetts, USA) and probed with the indicated primary antibodies and HRP-conjugated secondary antibodies.

### Gene expression analysis

We isolated total RNA from cells using a Tissue RNA Kit (Biomiga Corporation, San Diego, USA) following the manufacturer’s instructions. We synthesized cDNA from 500 ng total RNA using the PrimerScript RT Reagent Kit (Takara Corporation, Dalian, China) and quantified mRNA levels by qPCR using the SYBR Premix Ex Taq Kit (Takara Corporation, Dalian, China). We ran samples in technical triplicates, and the calculated mRNA levels of the genes of interest were normalized to GAPDH mRNA levels in the same samples using the 2^−∆∆CT^ method. The primers utilized in our investigation are listed in Supplementary Table 5.

### Cell migration and invasion assays

Cells were seeded in six-well plates, scratched with 10 µl pipet tips and cultured in 1× DMEM solution without serum. Then, the cells were washed three times with phosphate-buffered saline (PBS) and photographed by microscopy (Leica, Wetzlar, Germany) at 0 hours, 6 hours and 12 hours. The migration distance was measured by Image-Pro Plus software.

Cells were seeded in 24-well invasion chambers (BD Biosciences, New Jersey, USA) with a Matrigel-coated film insert (8 mm pore). The mixed solution was diluted to generate a 1× DMEM solution containing 10% serum. Two days later, cells on the bottom surface of the filter were subjected to staining with crystal violet for 15 min and then washed three times with PBS, and the cell number was counted under a microscope (Leica, Wetzlar, Germany).

### Human tissue specimens and immunohistochemical analysis

The experiment with human tissues was authorized by the Human Ethics Committee of Shanghai General Hospital, Shanghai Jiao Tong University School of Medicine (Shanghai, China). All subjects provided written informed consent. Patients with radiotherapy or chemotherapy treatment before surgery were excluded. Survival time was calculated from the date of surgery to the date of death or last follow-up. Tumor-node-metastasis staging was performed according to American Joint Committee on Cancer (AJCC)/Union for International Cancer Control (UICC) standards.

IHC analysis was performed as previously described [8]. The pathological types of paraffin-embedded slides were checked again by HE staining before IHC analysis for TSPAN8, EGFR and SOX9. A rabbit polyclonal anti-TSPAN8 antibody (1:100, Abcam, ab7007), rabbit monoclonal anti-EGFR antibody (1:100, Abcam, ab52894) and rabbit monoclonal anti-SOX9 antibody (1:400, CST, #82630) were used. A DAB Substrate Kit (Zsbio Commerce Store) was used according to the manufacturer’s instructions. The scores for staining frequency (0 = 0%, 1 = 1%, 2 = 2–10%, 3 = 11–30%, 4 = 31–70% and 5 = 71–100%) and intensity (0 = negative, 1 = week, 2 = moderate and 3 = strong staining) were used. A DAB Substrate Kit (Zsbio Commerce Store) was used according to the manufacturer = =negative, 1 were used. A DAB Substrate Kit (Zsbio Commerce Store) was used according to the manufacturer = week, 2 were used. A DAB Substrate Kit (Zsbio Commerce Store) was used according to the manufacturer = moderate and 3 were used. A DAB Substrate Kit (Zsbio Commerce Store) was used according to the manufacturer = strong staining) were summed to obtain an overall staining score (OSS). An OSS of 0–2 was deemed low, 3–5 was deemed moderate and 6–8 was deemed high. The results were scored by two pathologists blinded to the clinicopathologic data.

### Electromobility shift assay

Nuclear extracts from 10^7^ HeLa cells transfected with Flag-SOX9 plasmid were prepared, and SOX9 was purified by immunoprecipitation (IP) using a Flag antibody for EMSA as previously described [8, 57]. DNA probes were purchased from Sangon Biotech (Shanghai, China). EMSAs were performed by incubating samples with purified Flag-SOX9 protein in binding buffer with the Gel Shift Assay Systems kit (Promega, Wisconsin, USA) according to the manufacturer’s protocol. Following incubation, samples were loaded onto a 4.5% native acrylamide gel and electrophoresed for 30 min at 130 V. Gels were scanned using a ChemiDoc imager (Bio-Rad Laboratories, California, USA.)

### Xenograft tumor studies

The animal experiment was performed as previously described [58]. Twenty-four female Balb/c nude mice (5 weeks old) were divided into two groups (six mice per group): a group receiving SW1990 cells with overexpression of TSPAN8 and a group receiving SW1990 cells without overexpression of TSPAN8. A small left abdominal flank incision was made, the spleen was exteriorized, and the prepared cells (1 × 10^6^ cells/50 µl/mouse) were injected into the spleen with a 30-gauge needle. To prevent tumor cell leakage and bleeding, a cotton swab was held over the site of injection for 5 min. The blood vessels of the injected spleen were ligated, and the injected spleen was removed. The wound was sutured with 6–0 black silk. Six weeks later, all of the mice were sacrificed and necropsied for observation of visible metastatic lesions in the liver. All animal experiments were approved by the animal care and use committee of Shanghai Renji Hospital, Shanghai Jiaotong University School of Medicine.

### Statistical analysis

Differences between groups were analyzed using Student’s *t* test, chi-square tests or Fisher’s exact test with GraphPad Prism 7.0 and SPSS 17.0 software. Pearson’s test was applied to determine the correlation between clinicopathological parameters and protein expression. Data are presented as the mean ± SD. Differences at *P* < 0.05 were considered statistically significant.

## Supplementary information

Supplementary figure 1

Supplementary figure 2

Supplementary figure 3

Supplementary figure 4

Supplementary figure 5

Supplementary table 1

Supplementary table 2

Supplementary table 3

Supplementary table 4

Supplementary table 5

Supplementary figure legends

Supplementary table legends

## Data Availability

All relevant data supporting the findings of this study are available from the corresponding author upon reasonable request.
