# Supplementary figures and images for: SOX9 is a critical regulator of TSPAN8-mediated metastasis in pancreatic cancer
Source: Oncogene. 2021 Jun 23;40(30):4884–93. doi: 10.1038/s41388-021-01864-9 (PMC8321899; doi:10.1038/s41388-021-01864-9)

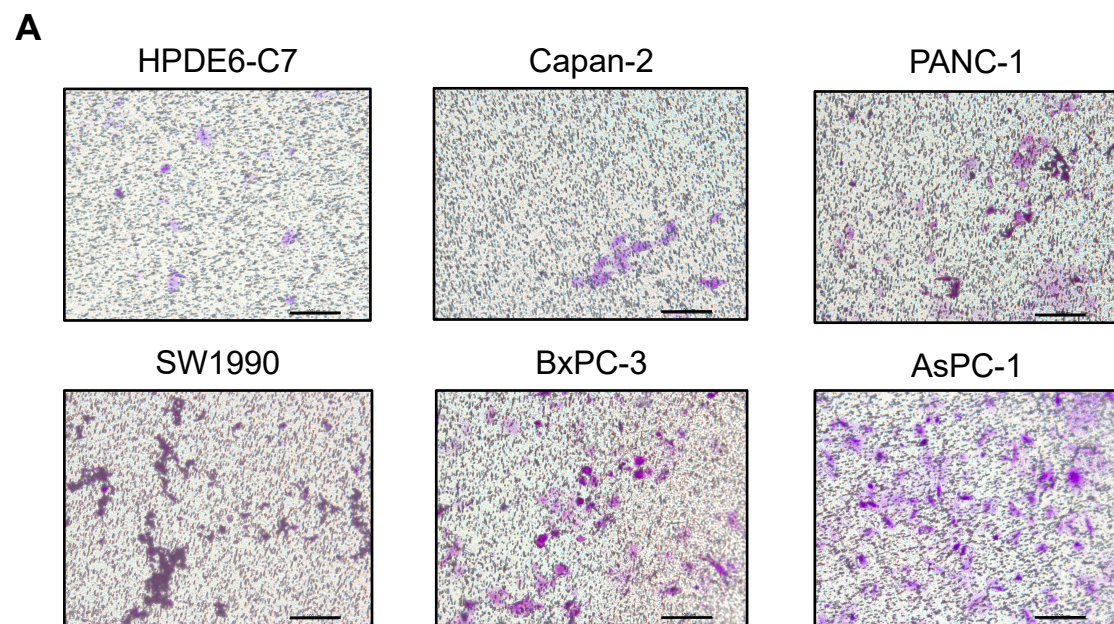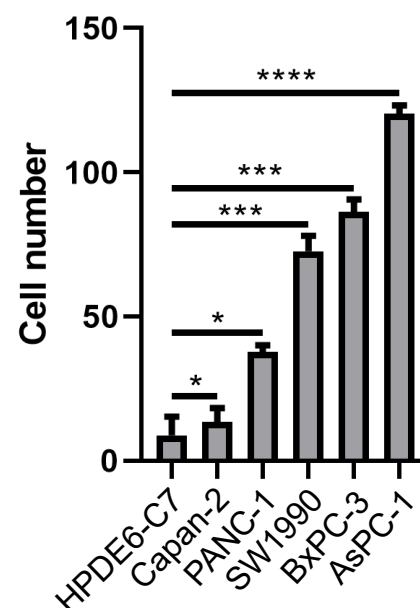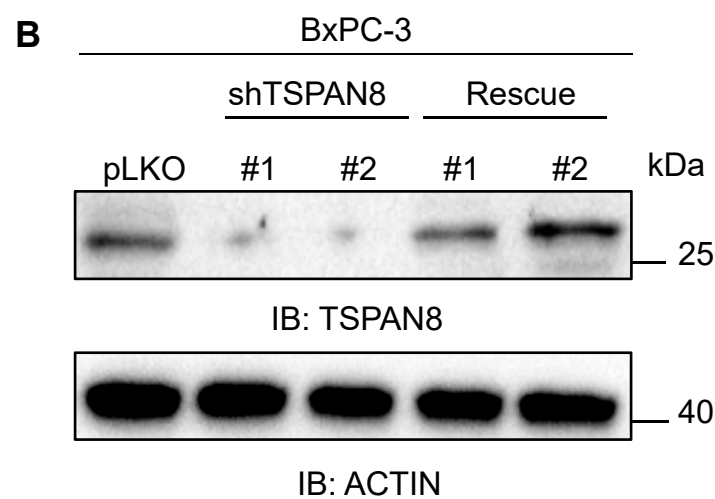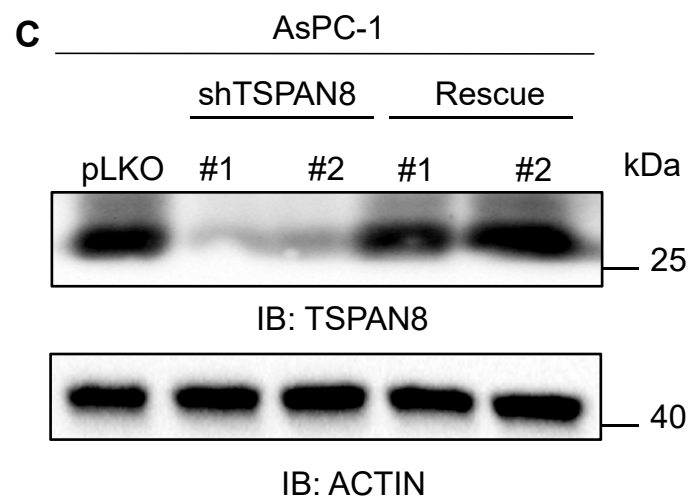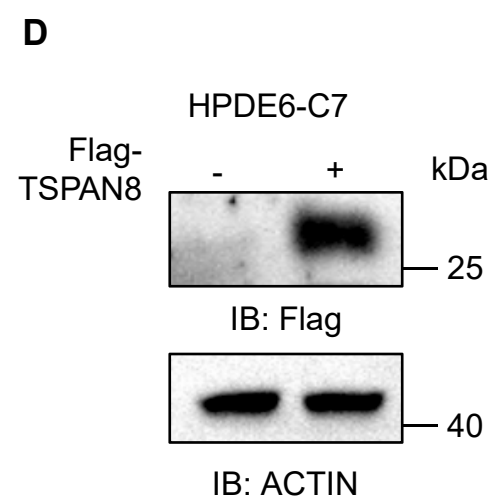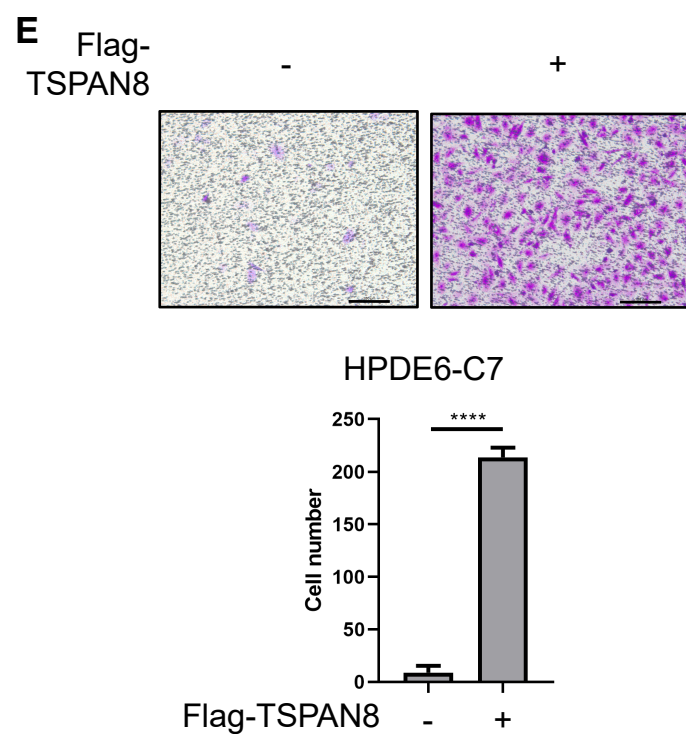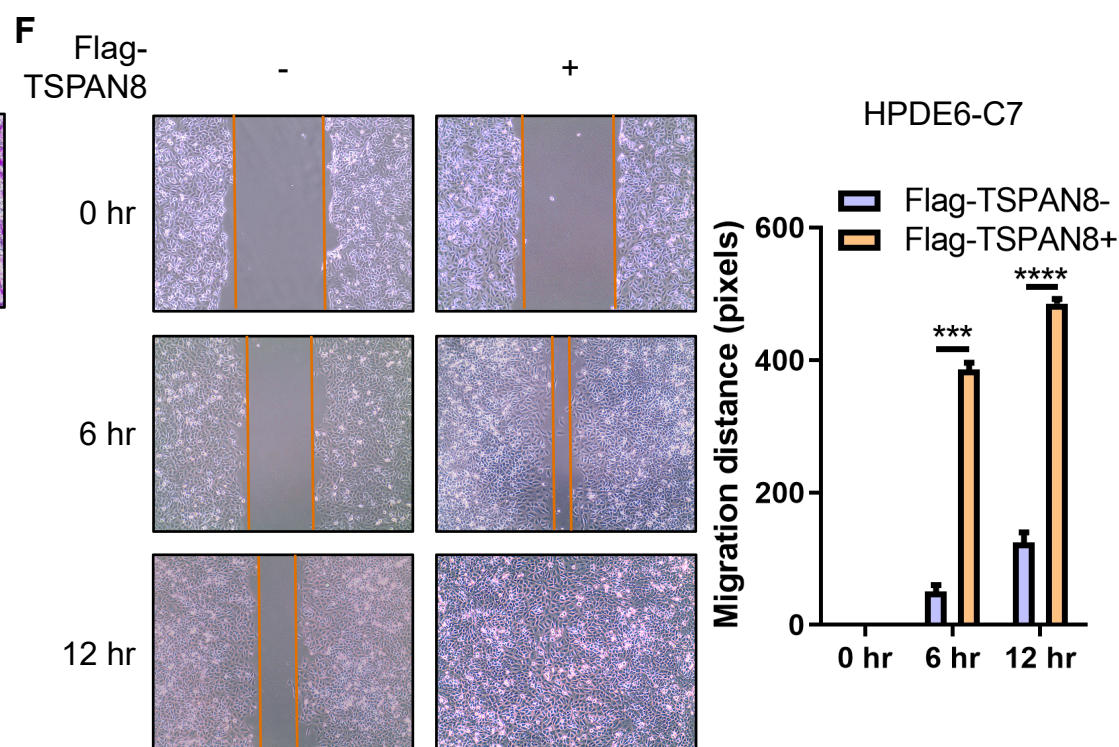

Supplement: Supplementary file 2 — Supplementary figure 2 [file 41388_2021_1864_MOESM2_ESM.pdf]

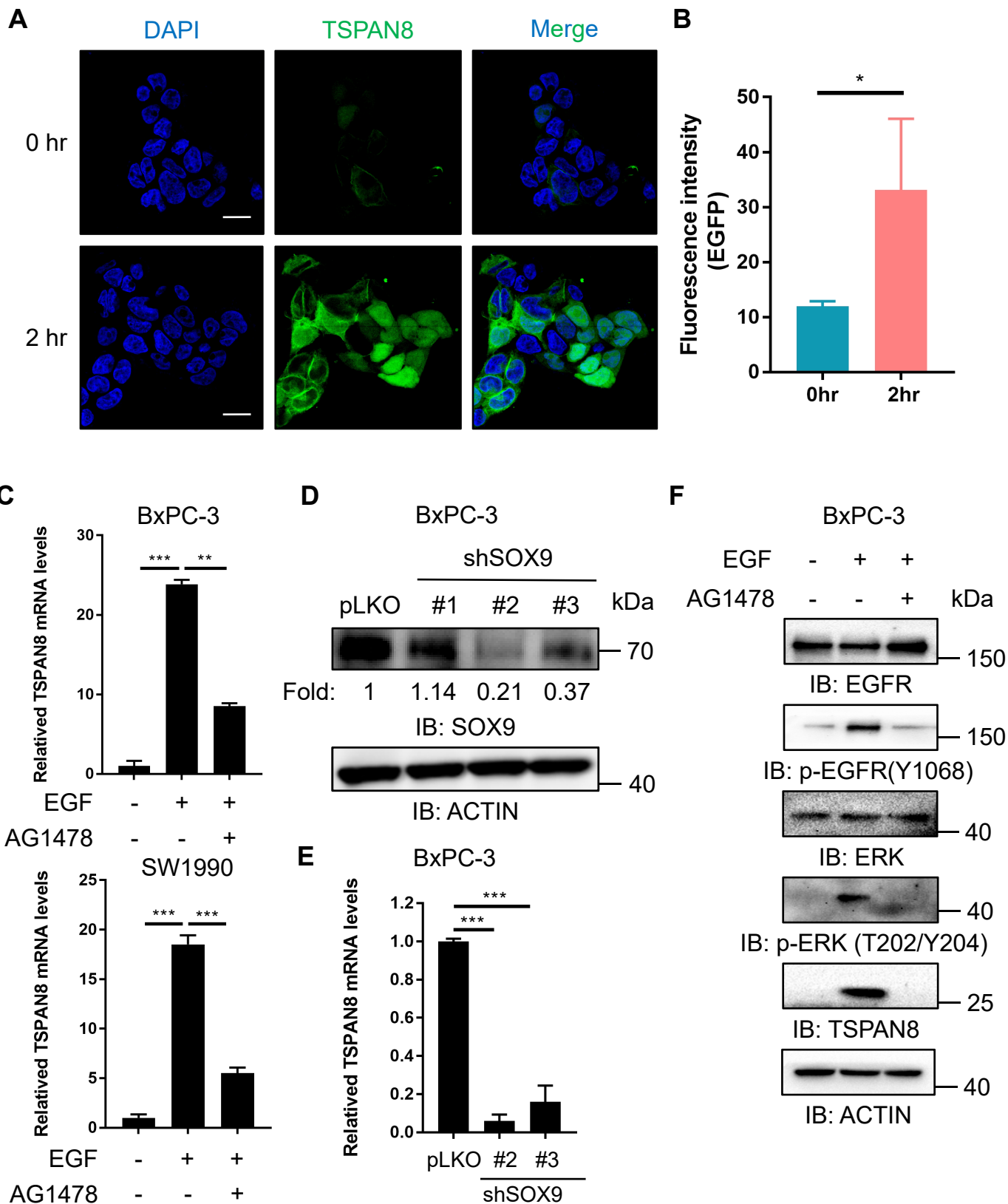

Supplement: Supplementary file 3 — Supplementary figure 3 [file 41388_2021_1864_MOESM3_ESM.pdf]

A

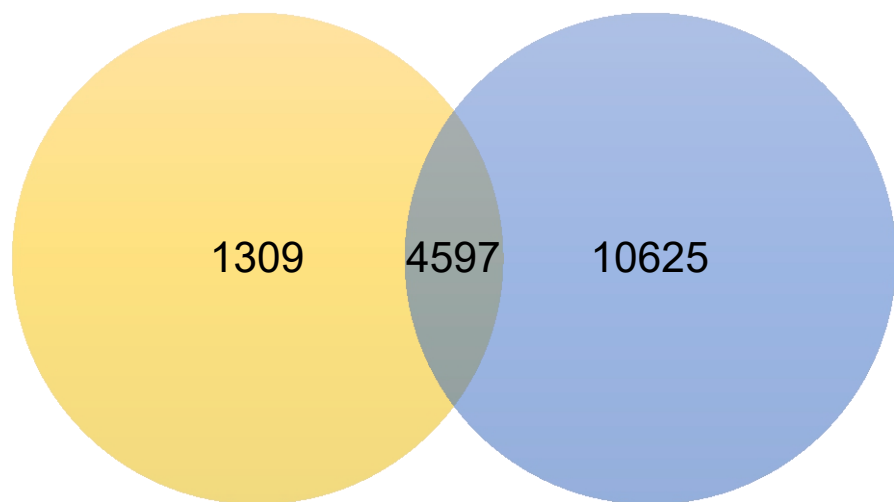

B

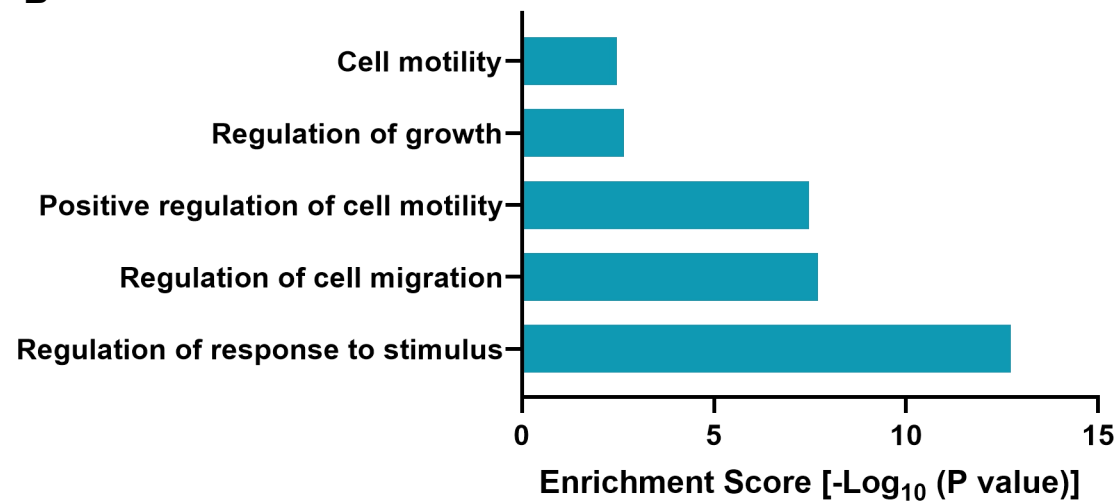

C

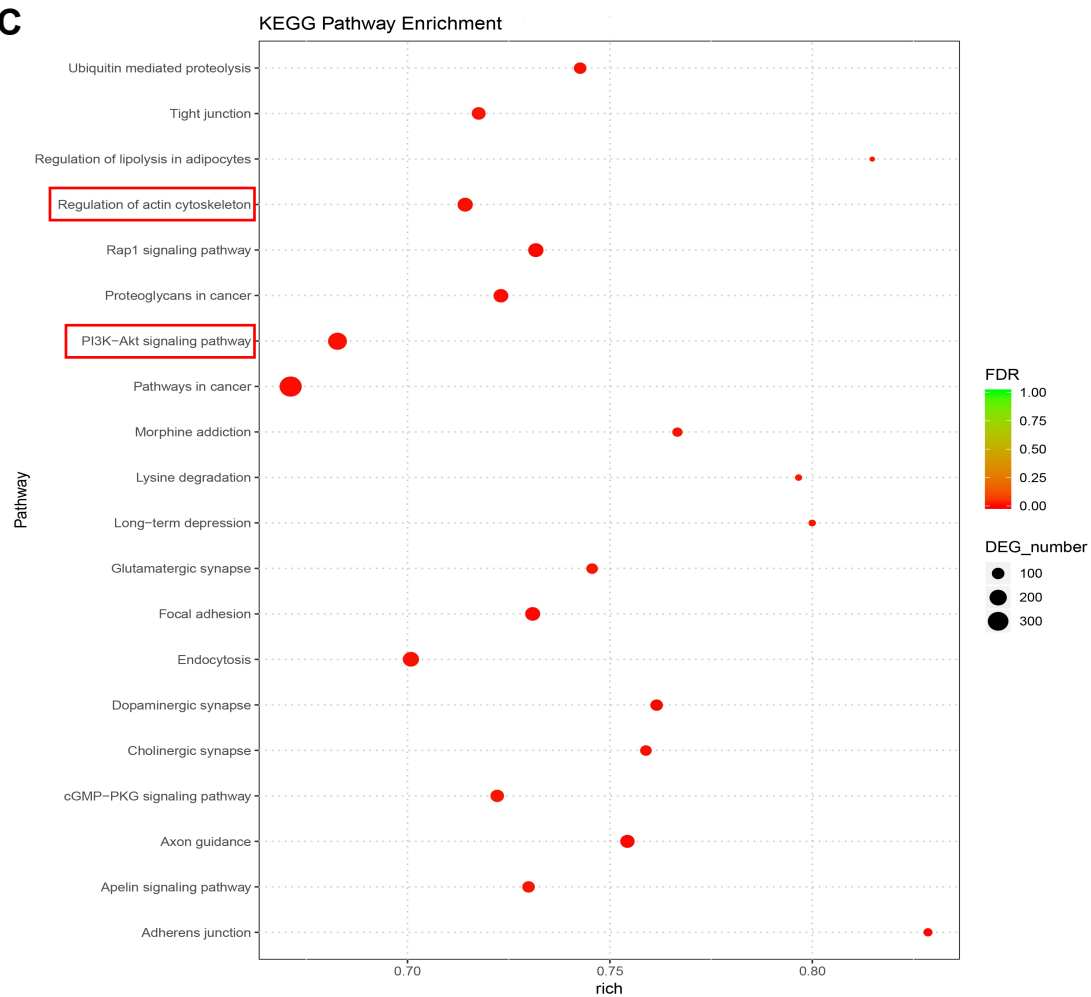

D

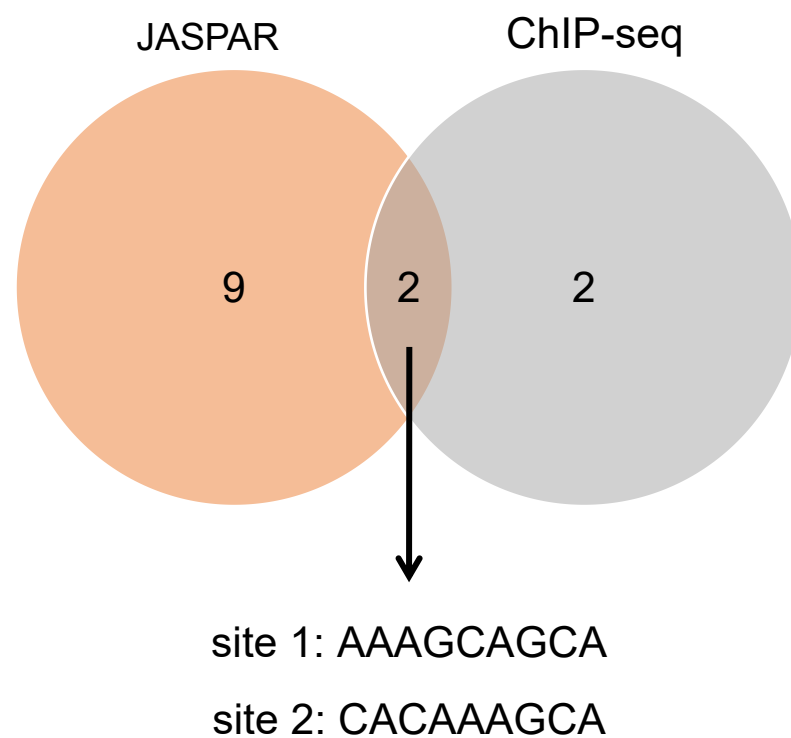

Supplement: Supplementary file 4 — Supplementary figure 4 [file 41388_2021_1864_MOESM4_ESM.pdf]

**A****Lung Cancer**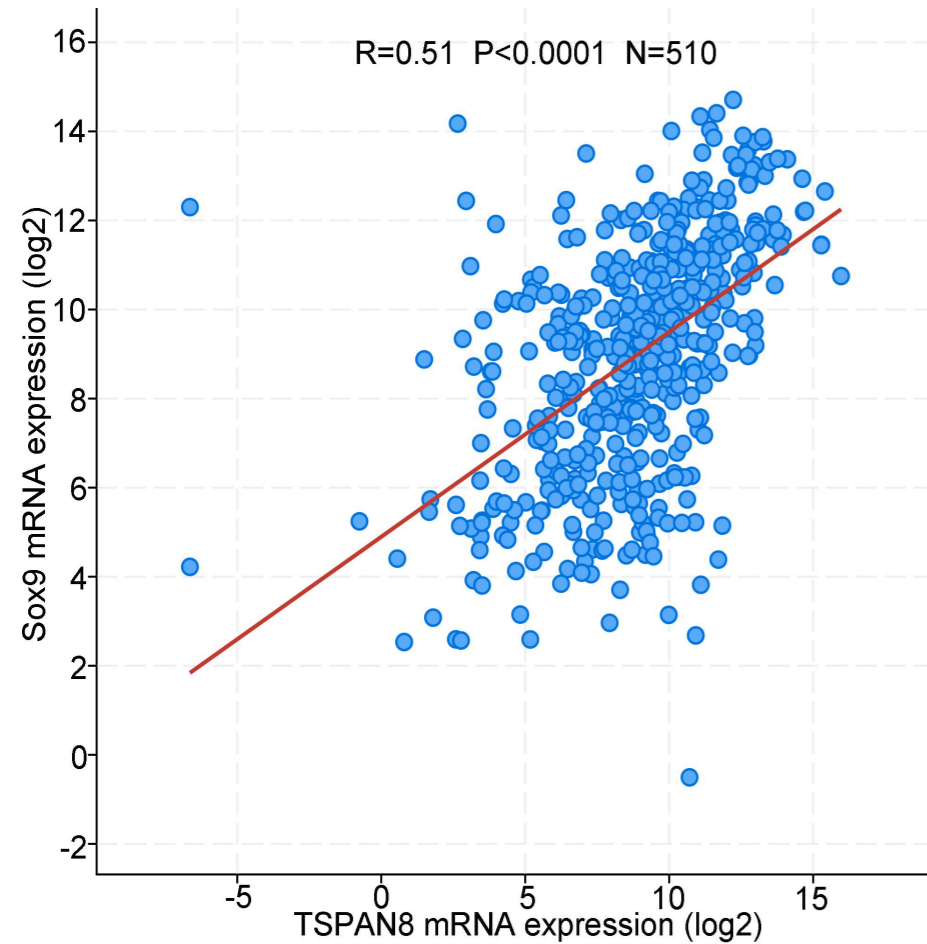**B****Prostate Cancer**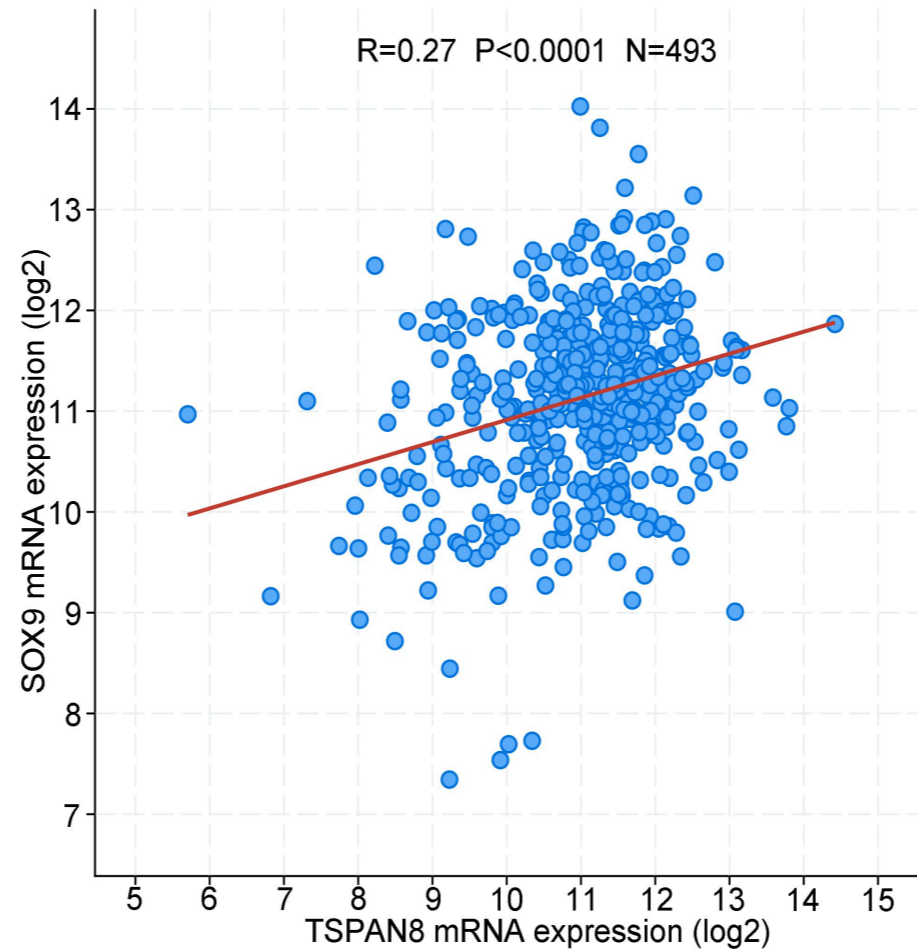**C****Skin Cancer**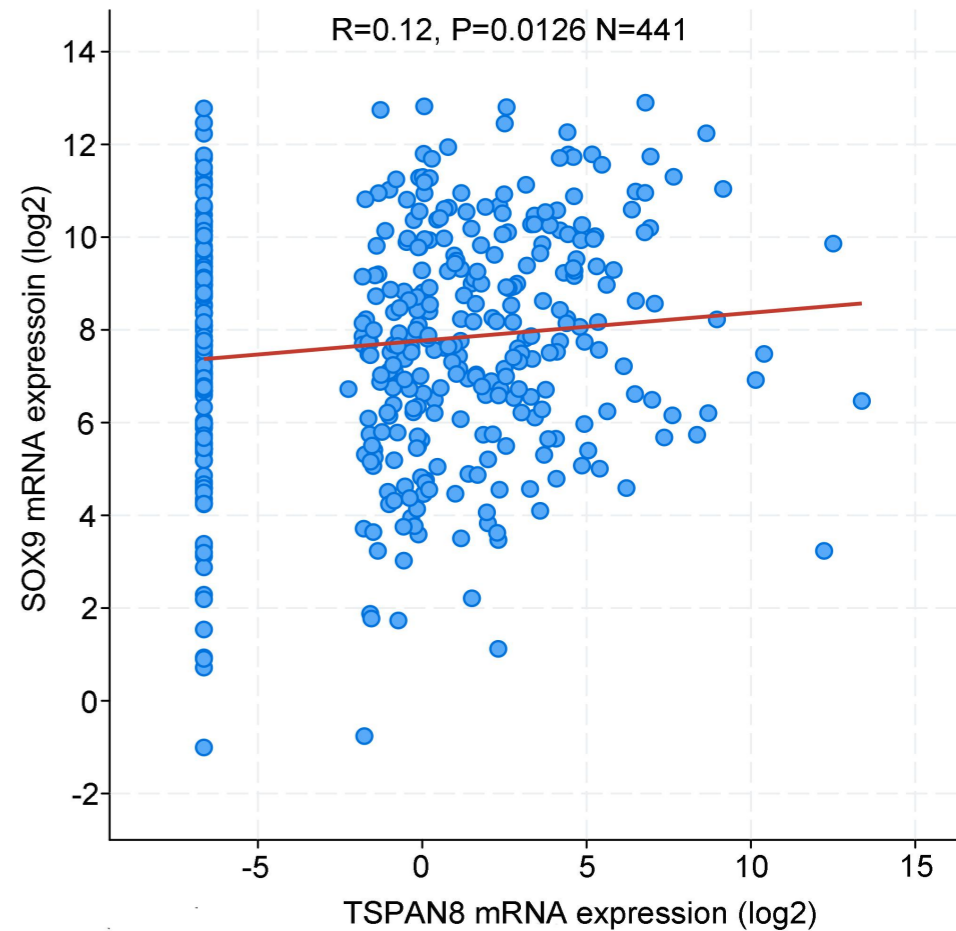

Supplement: Supplementary file 5 — Supplementary figure 5 [file 41388_2021_1864_MOESM5_ESM.pdf]
