# Supplementary material for: SOX9 is a critical regulator of TSPAN8-mediated metastasis in pancreatic cancer
Source: Oncogene. 2021 Jun 23;40(30):4884–93. doi: 10.1038/s41388-021-01864-9 (PMC8321899; doi:10.1038/s41388-021-01864-9)
Supplement: Supplementary file 11 — Supplementary figure legends [file 41388_2021_1864_MOESM11_ESM.docx]

**Supplementary Figure 1. TSPAN8 is highly expressed in multiple tumours and promotes tumour metastasis in mice.** (A-B) Immunohistochemical staining for TSPAN8 was performed on 15 human breast cancer (A) and 10 human hepatocellular carcinoma (B) specimens. Representative images of normal adjacent tissues (NATs) and tumour tissues (TTs) are shown. Scale bars: 200 μm. The t-test was performed to compare differences between tumour and normal adjacent tissues. (C) The *TSPAN8* mRNA level was analysed in TCGA and GTEx data via the GEPIA online tool (<http://gepia.cancer-pku.cn>). (D-E) SW1990 cells with or without expression of HA-TSPAN8 were injected into 6-week-old nude mice. The tumour metastasis ability was analysed.

**Supplementary Figure 2. TSPAN8** **is depleted by shRNA and rescued by complementation with shRNA-resistant TSPAN8 in BxPC-3 and AsPC-1 cells.** (A) The metastatic potential of the different pancreatic cell lines was quantified by Boyden chamber Matrigel invasion assays. A total of 5000 cells were seeded in 24-well invasion chambers. After 48 hr, the cells were counted under a microscope. BxPC-3 (B) and AsPC-1 (C) cells were expressed the control shRNA or *TSPAN8* shRNA vector or reconstituted with shRNA-resistant TSPAN8, and immunoblot analysis was performed with the TSPAN8 antibodies. (D-F) TSPAN8 was transiently overexpressed in HPDE6-C7 cells. Immunoblot analysis was performed with the TSPAN8 antibodies. Boyden chamber Matrigel invasion assays (E) and a wound healing assay (F) were performed. In A, E and F, the experiments were performed in triplicate, and the t-test was performed. Scale bars: 100 μm. The values are presented as the means ± SDs. * *P* <0.05, ** *P* <0.01, *** *P* <0.001, and **** *P* <0.0001.

**Supplementary Figure 3. EGF upregulates TSPAN8 expression.** (A-B) SW1990 cells expressing TSPAN8-EGFP were treated with 100 ng/ml EGF for 2 hr. Immunofluorescence analysis was performed (A). The fluorescence intensity of EGFP was calculated (B). (C) BxPC-3 and SW1990 cells were treated with or without AG1478 (10 μM) for 1 hr prior to EGF treatment (100 ng/ml) for 2 hr. (D) BxPC-3 cells expressing the control shRNA or SOX9 shRNA vector were subjected to immunoblot analysis. (E) TSPAN8 mRNA levels were analysed by q-PCR in BxPC-3 cells with or without SOX9 shRNA expression. (F) BxPC-3 cells were treated with or without AG1478 (10 μM) for 1 hr prior to EGF treatment (100 ng/ml) for 2 hr. Protein expression was analysed by immunoblotting with the indicated antibodies. In B, C and E, the experiments were performed in triplicate, and the t-test was performed. The values are presented as the means ± SDs. * *P* <0.05 and *** *P* <0.001.

**Supplementary Figure 4. SOX9 regulates the expression of cell migration-related genes.** (A) Venn diagram showed the overlap of peaks in SW1990 cells stable overexpression of Flag-SOX9 (blue) or vector (yellow). (B) GO enrichment analysis showed the GO terms enriched of the different genes. (C) KEGG pathway enrichment analysis was performed with different genes. (D) Venn diagram showed the overlap of binding sites of SOX9 in the promoter region of *TSPAN8.*

**Supplementary Figure 5. Correlation analysis of *SOX9* and *TSPAN8* mRNA expression in lung cancer, prostate cancer and skin cancer.** Correlation analysis of *SOX9* and *TSPAN8* mRNA expression in lung cancer (A), prostate cancer (B) and skin cancer (C) were performed with data in the TCGA dataset.
