# Supplementary material for: SOX9 is a critical regulator of TSPAN8-mediated metastasis in pancreatic cancer
Source: Oncogene. 2021 Jun 23;40(30):4884–93. doi: 10.1038/s41388-021-01864-9 (PMC8321899; doi:10.1038/s41388-021-01864-9)
Supplement: Supplementary file 12 — Supplementary table legends [file 41388_2021_1864_MOESM12_ESM.docx]

**Supplementary Table 1. TSPAN8 IHC staining in normal adjacent tissues and tumor tissues in PDAC.**

**Supplementary Table 2. The GO terms of SOX9 enriching genes response to stimulus, cell migration, motility and growth.**

**Supplementary Table 3.** **The GO terms which P<0.05 in our research.**

**Supplementary Table 4. The shRNA and PCR primer oligonucleotides sequences in our research.**

**Supplementary Table 5. The qPCR primer in our research.**
