# Supplementary material for: SOX9 is a critical regulator of TSPAN8-mediated metastasis in pancreatic cancer
Source: Oncogene. 2021 Jun 23;40(30):4884–93. doi: 10.1038/s41388-021-01864-9 (PMC8321899; doi:10.1038/s41388-021-01864-9)

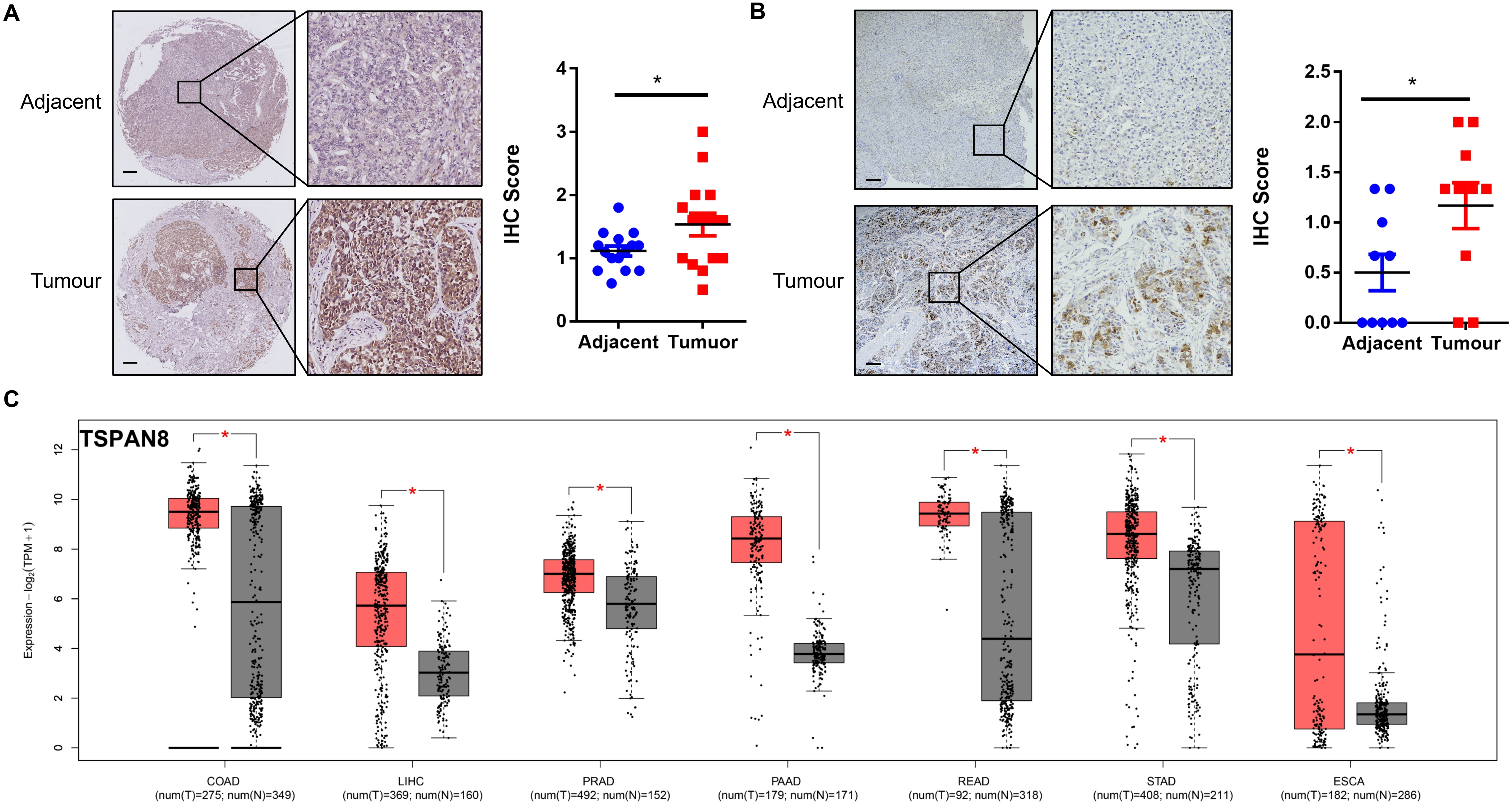

D

Tumorigenicity of implanted SW1990 (10^6)	
Group	Number of mice with metastasis
Vector	3/6
HA-TSPAN8	6/6

E

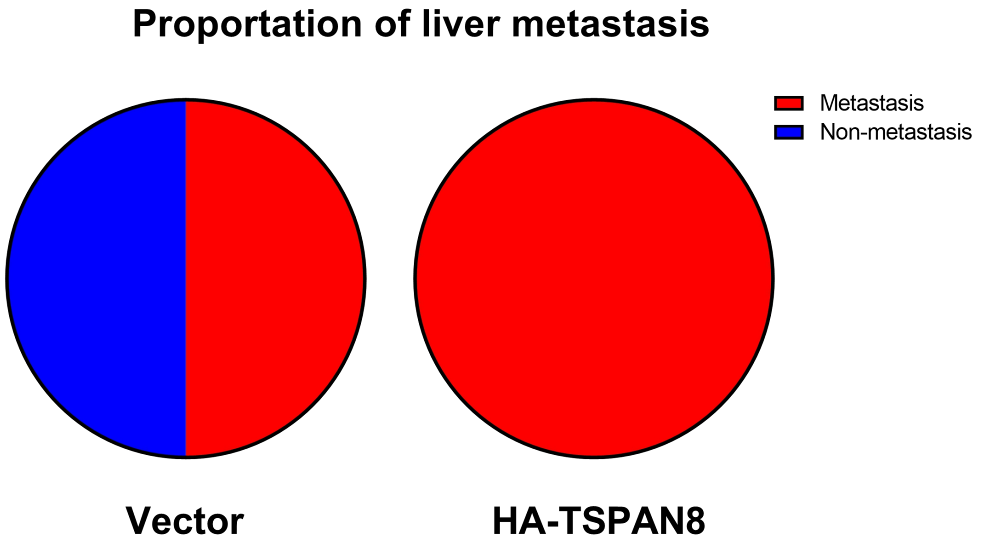

Supplement: Supplementary file 1 — Supplementary figure 1 [file 41388_2021_1864_MOESM1_ESM.pdf]
